# Sinus of Valsalva Aneurysm: A Rare Cause of Dyspnea

**DOI:** 10.1155/2015/467935

**Published:** 2015-08-24

**Authors:** Aiman Smer, Osama Elsallabi, Mohamed Ayan, Haitam Buaisha, Hamza Rayes, Yazeid Alshebani, Hamza Tantoush, Mohsin Salih

**Affiliations:** Department of Cardiovascular Medicine, Creighton Cardiac Center, Creighton University School of Medicine, 3006 Webster Street, Omaha, NE 68131, USA

## Abstract

Sinus of Valsalva aneurysm (SOVA) is a rare clinical entity. Clinical manifestations can vary from an incidental finding on an imaging study to a life-threatening emergency. We report a case of a 51-year-old female with a large symptomatic left SOVA. Echocardiogram and computed tomography angiography (CTA) of the chest revealed marked dilatation of the left sinus of Valsalva, measuring 7.5 cm. This resulted in superior displacement of the left main coronary artery. Surgical repair of the aneurysm with reimplantation of the right and left coronary arteries was performed in addition to aortic valve replacement (Bentall procedure). The patient had an uneventful postoperative course and remains asymptomatic at the three-month follow-up visit.

## 1. Introduction

A left sinus of Valsalva aneurysm is the rarest of all Valsalva sinus aneurysms. However, it is critical to recognize that this defect can be a cause of acute heart failure. To the best of our knowledge, there have been only 30 cases of left SOVA reported in the literature. The majority of these reports have described ruptured SOVAs, most commonly occurring in the right ventricle. Small and unruptured left SOVAs are clinically silent and often discovered incidentally on imaging study. SOVAs can be associated with aortic regurgitation (AR), ventricular septal defects (VSD), and infective endocarditis. Echocardiography may demonstrate a classic “windsock” appearance at the aortic short axis view. Surgical repair is the treatment of choice for ruptured SOVAs. We report a case of a large symptomatic unruptured left SOVA with superior displacement of the left main coronary artery which required urgent aneurysmal repair.

## 2. Case Presentation

**Figure 1:**
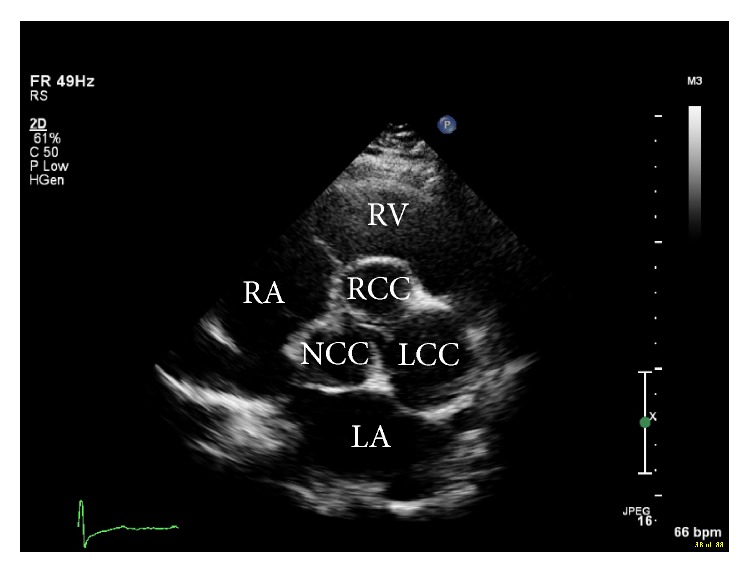
Parasternal short axis view of the aortic valve shows dilated left sinus of Valsalva. Note that the left coronary cusp (LCC) is markedly enlarged compared to the other two cusps. Normally, all three cusps are equal in size. RV: right ventricle, RA: right atrium, and LA: left atrium. RCC: right coronary cusp; NCC: noncoronary cusp.

**Figure 2:**
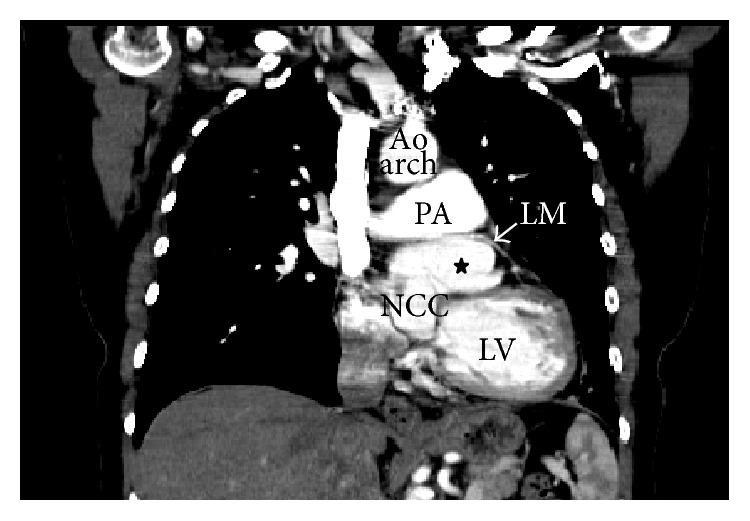
Coronal section of the chest CT shows markedly enlarged left sinus of Valsalva (asterisk). Also note that the course of the left main (LM) coronary artery is displaced superiorly and passing through the dilated sinus of Valsalva and main pulmonary artery (PA). NCC: noncoronary cups, LV: left ventricle, and Ao arch: aortic arch.

**Figure 3:**
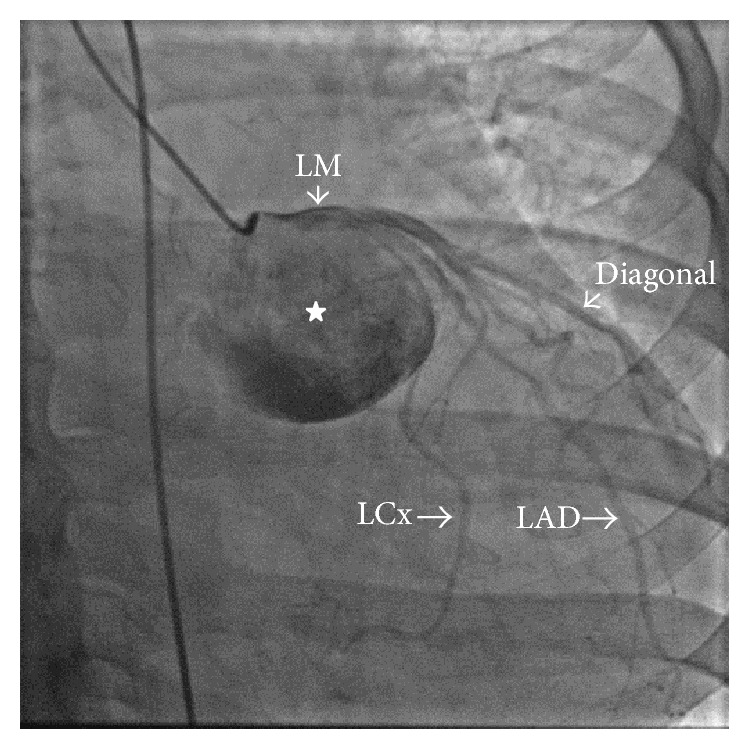
Coronary angiogram shows contrast filling the enlarged left sinus of Valsalva (asterisk). LM: left main coronary artery, LAD: left anterior descending artery, and LCx: left circumflex artery.

An otherwise healthy 51-year-old Caucasian female presented with acute onset shortness of breath. She was unable to walk more than a few blocks. She denied any symptoms of chest pain or palpitations and had no family history of cardiac or familial disorders. Vitals revealed a blood pressure of 140/65 mmHg, pulse of 85 bpm, respiratory rate of 18, and temperature of 98.4°F. Cardiac examination revealed normal S1, soft S2, and grade II/VI early diastolic murmur in the left parasternal area. No jugular venous distention or pedal edema was noted. The remainder of the exam was unremarkable. Laboratory studies were within normal range. The resting electrocardiogram showed sinus rhythm with frequent premature atrial complexes. There were no ST-T wave changes. Transthoracic echocardiogram (TTE) revealed normal left ventricular size and function with ejection fraction of 55–60%, moderate aortic root dilatation, and aneurysmal dilatation of the left sinus of Valsalva (Figure 1). There was moderate AR and mild mitral regurgitation (Video 1 in Supplementary Material available online at http://dx.doi.org/10.1155/2015/467935). CTA revealed marked dilatation at the level of the left sinus of Valsalva, measuring 7.5 cm (Figure 2). Coronary angiography demonstrated superiorly displaced left main coronary artery with no significant stenosis or obstruction noted (Figure 3) (Video 2). Because the patient was symptomatic and had concomitant significant aortic regurgitation, surgery was indicated. Surgical exploration of the chest revealed a massively dilated left sinus of Valsalva with the left main coronary artery arising from the superior aspect. The patient underwent replacement of the aortic valve with a size #27 St. Jude mechanical prosthesis and Dacron conduit, with reimplantation of the right and left coronary arteries on the anterior and lateral surfaces, respectively (Bentall procedure). The postoperative course was uneventful. She remains asymptomatic at the three-month follow-up visit. Repeat echocardiography shows no residual abnormalities.

## 3. Discussion

SOVA is a rare but serious cardiac anomaly. It was first described by Hope in 1839 [1]. Incidence varies from 0.1 to 3.5% among all congenital cardiac lesions [2]. The left SOVA is the rarest of all SOVAs (<5%) [3]. SOVA can be congenital or acquired. The right coronary sinus is by far the most common site of origin for congenital SOVAs. These are usually associated with other congenital cardiac defects. VSD and AR are the most common coexisting cardiac defects. Acquired aneurysms are mostly caused by trauma, atherosclerosis, endocarditis, syphilis, and connective tissue diseases [2]. Most SOVAs result from a congenital deficiency of elastic lamellae in the wall of the affected sinus, with separation of the media in the sinus of Valsalva from the media adjacent to the aortic valve annulus [4]. Eventually, this weak area enlarges and becomes aneurysmal due to the effect of high aortic pressure. Clinical manifestations may vary from an incidental finding on an imaging study to a life-threatening emergency. Unruptured SOVAs typically remain asymptomatic for decades and are often discovered incidentally on echocardiography or other imaging modalities. However, large unruptured aneurysms can produce mass effect and may cause valvular dysfunction, right ventricular outflow tract obstruction, and myocardial ischemia due to compression on the origin of the main coronary arteries [5]. Nonetheless, acute symptoms usually occur in most cases of aneurysmal rupture and subsequent fistula formation to the adjacent cardiac chamber. Acute symptoms consist of sudden shortness of breath and chest pain. Moreover, a characteristic loud continuous murmur accompanied with a thrill is often related to rupture of the aneurysm [6]. Congenital SOVAs usually rupture during the third and fourth decade of life. Generally, aneurysms rupture into the right ventricle followed by right atrium. In contrast, left aneurysms rupture into the pericardial cavity. Other potential complications of SOVAs include AR, myocardial ischemia, conduction abnormalities, and thrombus formation with subsequent systemic embolization [7]. Nonetheless, heart failure is still the main cause of death in patients with SOVAs. Echocardiography is very useful in the diagnosis of SOVAs and associated lesions such as AR, VSD, and endocarditis [8]. Other diagnostic modalities such as CTA, cardiac MRI, and cardiac catheterization with aortography are often necessary to confirm diagnosis, identify coronary anatomy, and detect complications. Surgical repair is the treatment of choice for ruptured SOVAs with excellent long term survival of 97.3% at 10 years [9, 10]. An unruptured but symptomatic SOVA should also be considered for surgical intervention. Moreover, concomitant VSD, endocarditis, and moderate to severe aortic valve regurgitation require surgery [11]. Conversely, several reports have shown that percutaneous closure of ruptured SOVA using transcatheter closure devices is feasible and effective therapeutic alternative to surgery [12–14].

## 4. Conclusion

Left sinus of Valsalva aneurysm is a very rare but serious cardiac defect. Rupture of SOVA is the most fearful complication. Physicians should consider the diagnosis of rupture of SOVA in young healthy patients who present with acute onset of shortness of breath and were found to have continuous murmur on physical examination. Echocardiography is the most useful diagnostic tool for diagnosis of SOVA and, in some cases, may demonstrate a classic “windsock” appearance at the aortic short axis view. Although surgical repair is considered, the treatment of choice for ruptured SOVA, percutaneous closure is an evolving therapeutic alternative.

## Supplementary Material

Video 1: Parasternal short axis view of the aortic valve. Left panel; shows markedly dilated left sinus of valsalva compared to the right and noncoronary cusps. Right panel; color flow Doppler demonstrates aortic regurgitant jet occupy > 25% of the left ventricular outflow tract area, which indicates moderate aortic regurgitation.
Video 2: In right anterior oblique projection, contrast injection shows superiorly displaced left main coronary artery as well as severely dilated left sinus of valsalva.
